# Ultrastructural characterization of peripheral denervation in a mouse model of Type III spinal muscular atrophy

**DOI:** 10.1007/s00702-021-02353-9

**Published:** 2021-05-17

**Authors:** Federica Fulceri, Francesca Biagioni, Fiona Limanaqi, Carla L. Busceti, Larisa Ryskalin, Paola Lenzi, Francesco Fornai

**Affiliations:** 1 Department of Clinical and Experimental Medicine, University of Pisa, Via Roma 55, 56126 Pisa, Italy; 2 I.R.C.C.S. Neuromed, Via Atinense 18, 86077 Pozzilli, IS Italy; 3 Department of Translational Research and New Technologies in Medicine and Surgery, University of Pisa, Via Roma 55, 56126 Pisa, Italy

**Keywords:** Neuromuscular disease, Muscle denervation, Muscle spindle, SMN, Transmission electron microscopy, Mitochondria

## Abstract

Spinal muscular atrophy (SMA) is a heritable, autosomal recessive neuromuscular disorder characterized by a loss of the survival of motor neurons (SMN) protein, which leads to degeneration of lower motor neurons, and muscle atrophy. Despite SMA being nosographically classified as a motor neuron disease, recent advances indicate that peripheral alterations at the level of the neuromuscular junction (NMJ), involving the muscle, and axons of the sensory-motor system, occur early, and may even precede motor neuron loss. In the present study, we used a mouse model of slow progressive (type III) SMA, whereby the absence of the mouse SMN protein is compensated by the expression of two human genes (heterozygous *SMN1A2G*, and *SMN2*). This leads to late disease onset and prolonged survival, which allows for dissecting slow degenerative steps operating early in SMA pathogenesis. In this purely morphological study carried out at transmission electron microscopy, we extend the examination of motor neurons and proximal axons towards peripheral components, including distal axons, muscle fibers, and also muscle spindles. We document remarkable ultrastructural alterations being consistent with early peripheral denervation in SMA, which may shift the ultimate anatomical target in neuromuscular disease from the spinal cord towards the muscle. This concerns mostly mitochondrial alterations within distal axons and muscle, which are quantified here through ultrastructural morphometry. The present study is expected to provide a deeper knowledge of early pathogenic mechanisms in SMA.

## Introduction

Spinal muscular atrophy (SMA) is a heritable, autosomal recessive neuromuscular disorder, which encompasses a broader group of disease subtypes all sharing loss-of-function mutations/conversion or deletion in the survival of motor neurons 1 (*SMN1*) gene (Lefebvre et al. 1995; Arnold et al. 2015). These homozygous disruptions lead to a deficit in the ubiquitous SMN protein, which is known to regulate RNA processing, mostly small nuclear ribonucleoprotein (snRNP) biogenesis and pre-mRNA splicing (Li et al. 2014). This occurs mostly during neuronal development when SMN protein localizes predominantly in the nucleus within “gems” or Cajal bodies (Liu and Dreyfuss 1996; Hebert et al. 2001; Navascues et al. 2004). During neuronal maturation, a progressive shift in SMN localization from the nucleus towards the cytoplasm and axoplasm occurs (Giavazzi et al. 2006). SMN localization within dendrites and axons of motor neurons, and also in peripheral components (e.g. muscle), suggested additional roles aside from its canonical functions in the spliceosome (Pagliardini et al. 2000; Rajendra et al. 2007). As recently reviewed, SMN plays a more general housekeeping role by intermingling with various, ubiquitous cell processes. These include RNA translation, cytoskeletal dynamics and endocytosis, autophagy and ubiquitin–proteasome cell-clearing pathways, as well as mitochondrial activity and bioenergetics (Chaytow et al. 2018).

The loss of SMN protein due to homozygous disruptions of *SMN1* leads to progressive degeneration of lower motor neurons (MNs) associated with muscle atrophy and paralysis. However, the age of onset, clinical phenotype, and degree of severity vary among four different (type I-IV) SMA subtypes (Zerres and Rudnik-Schoneborn 1995; Lunn and Wang 2008; Arnold et al. 2015). Type I, also known as Werdnig-Hoffmann disease, is a very severe form with very early onset before the age of 6 months, rep-resenting an overall 45% of SMA cases, and the most common genetic cause of infant mortality within 2 years of life (Zerres and Rudnik-Schoneborn 1995; Lunn and Wang 2008; Arnold et al. 2015). Type II, or Dubowitz disease, is an intermediate SMA form, with onset between 7 and 18 months. Generally, the ability to stay seated independently is preserved and survival is into adulthood, except for cases in which respiratory compromise due to restrictive lung disease may occur (Zerres and Rudnik-Schoneborn 1995; Lunn and Wang 2008; Arnold et al. 2015). Type III SMA, also known as Kugelberg and Welander syndrome, is a slowly progressing form with onset after 30 months of life, with patients typically having normal milestones in the first year of life. Generally, ambulation is preserved over many years, and the prognosis is good (Zerres and Rudnik-Schoneborn 1995; Lunn and Wang 2008; Arnold et al. 2015). Finally, type IV SMA, which may occur an as autosomal dominant disorder, is the less severe subtype, with an onset between 10 and 30 years (Monani 2005). Despite a limb-girdle phenotype, it allows patients to have a normal lifespan (Mercuri et al. 2012; Arnold et al. 2015). The milder (type II-IV) SMA phenotypes are in part associated with an increase in the (dosage) copy number of the *SMN2* gene, which codes for a centromeric analog copy of SMN1 protein (Campbell et al. 1997; Farrar and Kiernan 2015). In fact, even the small amount of full-length transcript generated by *SMN2* may partly compensate for the loss of *SMN1*-produced protein, with *SMN1* dosage which correlates inversely with disease severity (Monani et al. 2000a; Arnold et al. 2015), which is in line with the piooner study of Lefebvre et al. (1997).

Despite recent advances, in-depth knowledge of the molecular mechanisms and fine neuropathology of SMA is still lacking. Since obstacles still exist with obtaining human specimens from either biopsy or post-mortem samples, research efforts aimed at validating appropriate SMA animal models are key. In 1997, the first SMA model featuring *SMN* knockout (KO) was generated by intercrossing (Smn+/−) mice. In this model, death occurs early at embryonic stages due to a failure to progress to the blastocyst stage (Schrank et al. 1997). Three years later, it was demonstrated that introducing the human *SMN2* gene in variable amount increases the life span in *SMN*-KO mice (Hsieh-Li et al. 2000; Monani et al. 2000b). Being reminiscent of what occurs in SMA patients, who carry at least one or more copies of the *SMN2* gene, these mice express, with high variability, motor deficit and spinal cord pathology, which is characterized by degenerating MNs and muscle denervation (Hsieh-Li et al. 2000; Monani et al. 2000b). Increasing *SMN2* dosage further attenuates the motor neuron disorder and prolongs survival, allowing post-natal life in such a model (Monani et al. 2000a, b). Still, the disease has an early onset with severe motor impairment and a short life-span, which led to propose this model as reminiscent of type I human SMA (Monani et al. 2000b). Nonetheless, the lack of a sufficient time window, which could instead allow for dissecting potential degenerative phenomena operating early in SMA pathogenesis, added a further level of complexity in animal SMA research. In an effort to obtain animal models with longer survival and slower disease progression, which could better mimic type III human SMA, a novel *SMN*-KO (Smn−/−) mouse model was generated featuring a human *SMN1* mutation (*SMN1A2G*), along with human *SMN2* (Monani et al. 2003). Contrarily to homozygous *SMN1A2G* mice, which do not feature motor alterations, heterozygous *SMN1A2G* mice develop a slow-progressive motor neuron loss, which is reminiscent of human SMA III (Monani et al. 2003; Gavrilina et al. 2008). In fact, in the present model, the absence of the mouse SMN protein is compensated by the expression of two human genes (heterozygous *SMN1A2G*, and *SMN2*), which leads to late disease onset and prolonged survival. This renders such a model quite different from most experimental models characterized by early and massive MN loss, which progresses rapidly in condensed time intervals. In fact, the short time window (a few weeks) of motor deterioration occurring in most SMA I models is likely to recruit molecular mechanisms that differ from those occurring during slowly progressive degeneration (lasting more than a year). This also applies to the occurrence of compensatory mechanisms (Monani et al. 2003; Fulceri et al. 2012).

In our previous studies, we used this knockout double transgenic (*SMN2*+/+; *Smn*−/− ; *SMN1A2G*+/−) SMA III mouse model to characterize the spinal cord pathology along with motor deficit at prolonged survival times (up to 535 days) (Fulceri et al. 2012; Biagioni et al. 2017). In particular, MN loss was quantified along with size variations of spared MNs, as well the occurrence of heterotopic MNs and radial glia within the white matter. This allowed to detail, for the first time, SMA III neuropathology at stereological level, which was carried out at long time intervals corresponding to almost 18 months. At this time point, a rough 40% of MNs loss was documented at a steady-state, when the deficit in motor activity did not progress any further. This consisted of a reduction in hind limb extension reflex and paw grip endurance, which started at 200 and 85 days, respectively, and reached a plateau at nearly 300 days of disease progression. Instead, the rota-rod and stride-length test outcomes were not altered at any time points (Fulceri et al. 2012; Biagioni et al. 2017). Such a dissociation in time and severity concerning MN loss and motor impairment suggests that other biological phenomena are involved in SMA pathogenesis, among which peripheral changes are taking center stage.

In fact, recent advances in SMA experimental research and clinics indicate that peripheral alterations at the level of the neuromuscular junction (NMJ), involving the muscle and distal axons, including sensory fibers, occur early, and may even precede MNs loss (Mentis et al. 2011; Bowerman et al. 2012; Wadman et al. 2012; Fayzullina and Martin 2014; Edens et al. 2015; Boido and Vercelli 2016; Fletcher et al. 2017; Vukojicic et al. 2019; Lefebvre and Sarret 2020). This is reminiscent of what is quite well-confirmed in other neuromuscular disorders such as Amyotrophic Lateral Sclerosis (ALS) and Spinal Bulbar Muscular Atrophy (SBMA) or Kennedy’s disease (Dupuis and Echaniz-Laguna 2010; Natale et al. 2015; Lalancette-Hebert et al. 2016; Limanaqi et al. 2017, 2020). Indeed, the role of NMJs in the pathogenesis of neuromuscular disorders is anything but peripheral, since early alterations involving components of the sensory-motor system, including the muscular endplate, muscle spindle, and proprioceptive fibers, may critically contribute to disease onset through muscle denervation and altered MNs excitability, up to MNs loss (Kararizou et al. 2006; Rajendra et al. 2007; Mentis et al. 2011; Boido and Vercelli 2016; Fletcher et al. 2017; Vukojicic et al. 2019). This is bound to (1) the lack of SMN protein, which besides MNs, is critical for the homeostasis of NMJ synapses, sensory and motor axons, and muscles, and (2) impairment of retrograde signals or transport mechanisms coming from NMJs (Rajendra et al. 2007; Bottai and Adami 2013; Boido and Vercelli 2016).

These data prompted us to extend the examination of motor neurons and proximal axons towards peripheral components, to unravel any potential ultrastructural alterations occurring in slowly progressive SMA. In the present study, we add to our previous observations in the *SMN*-KO double transgenic mouse model (*SMN2*+/+; *Smn*−/−; *SMN1A2G*+/−) by characterizing at ultrastructural level, the peripheral muscular denervation which is supposed to occur early in neuromuscular disorders, including SMA. In this purely morphological study carried out at transmission electron microscopy (TEM), we dissect the fine ultrastructure of muscles and distal axons in WT and SMA III mice, further extending our analysis to the muscle spindles. To our knowledge, this is the first report comprehensively documenting ultrastructural alterations within the muscle, muscle spindles, and distal axons in SMA III mice models. Remarkably, despite a 40% MNs loss and motor alterations characterizing these very same SMA mice, as assessed in our previous studies (Fulceri et al. 2012; Biagioni et al. 2017), the ultrastructure of surviving MNs and proximal axons is largely preserved. Instead, subcellular pathology within the muscle, distal axons, and muscle spindles appears mostly severe, with SMA muscles featuring a markedly disarranged sarcomere architecture, which is recapitulated by the severe alterations of intrafusal fibers occurring within the muscle spindle. Again, distal axons feature remarkable alterations in myelin sheath and clogging of axoplasm by abnormal, amorphous structures. These include intrusions of the myelin sheath itself, and electron-dense, amorphous material, including abnormal mitochondria with fragmentation and disappearance of cristae and ridges. In this frame, ultrastructural morphometry was applied to assess mitochondrial alterations, which turned out to be dramatic within the muscle and distal axons of SMA mice.

Our findings are consistent with a wide stream of evidence indicating peripheral denervation as a key event in the pathogenesis of neuromuscular disorders, which may shift the ultimate anatomical target in slow progressive SMA from the MNs within the spinal cord towards the muscle. This is expected to provide a platform for future experimental studies aimed at providing a deeper knowledge on the pathogenic mechanisms operating early in SMA, which could be key to fostering novel molecular targets and disease-modifying strategies.

## Materials and methods

### Animals

We used the KO, double transgenic mouse model (*N* = 10) carrying the genotype *Smn*−/−; *SMN1A2G*±; *SMN2*+/+ (SMA III mice) generated by the Jackson Laboratories (Bar Harbor, Maine, USA, Stock No. 5026). As control mice (*N* = 10) the FVB/NJ strain was used (Jackson Laboratory, Stock No. 1800), which corresponds to the Wild type (WT) for the KO double transgenic *Smn*-/-; *SMN1A2G* ±; *SMN2*+/+ mouse. This heterozygous mouse for the *SMN1A2G* gene owns barely detectable SMN protein levels compared with the homozygous strain (Monani et al. 2003), which was also confirmed by our previous studies (Fulceri et al. 2012). All experimental procedures were carried out according to the Guidelines of the European Council (86/609/EEC) for the use and care of laboratory animals. The experimental protocol was approved by the local Ethical Committee, and by the Ministry of Health.

Animals received food and water ad libitum and were housed under controlled conditions in 12 h light/dark cycle, and at 21 °C room temperature. Both WT and KO double transgenic (*Smn*−/−; *SMN1A2G*±; *SMN2*+/+) mice were killed at 18 months of age in order (1) to assess ultrastructural changes consistent with peripheral denervation in such a slowly progressive motor neuron disorder, and (2) not to risk further the occurrence of accidental deaths (cage deaths), which in longer time may have reduced mice number and/or bias the experimental findings. In fact, these experiments require an average of 2 years, making it difficult to replicate motor tests if the mice number is reduced. Motor tests and stereological motor neuron counts in these mice were previously performed and published by our group (Fulceri et al. 2012; Biagioni et al. 2017). For the present analysis, stored replicates from these previous studies (*N* = 10 mice per group) were selected.

### Tissue dissection and processing for transmission electron microscopy

Mice were deeply anesthetized with chloral hydrate and perfused trans-cardially with saline solution (0.9% NaCl) and the fixing solution 2% paraformaldehyde/0.1% glutaraldehyde in 0.1 M phosphate-buffered saline, pH = 7.4. The spinal cord and gastrocnemius muscle were dissected and moved overnight at 4 °C in the same fixing solution (2.0% paraformaldehyde and 0.1% glutaraldehyde in 0.1 M PBS, pH = 7.4). The lumbar tract of the spinal cord and gastrocnemius muscle were surgically dissected, with muscles being gently stretched for 10 s, and spinal cords being gently removed to avoid any abnormal pressure. Specimens were then immersed for 1 h and 30 min in the fixing solution used for perfusion. Afterward, specimens were post-fixed in a 1% OsO_4_ buffered solution for 1 h and 30 min at 4 °C, and then dehydrated in increasing ethanol solutions, and finally embedded in epoxy resin.

For each spinal cord sample, two tissue blocks (volume of 5 mm^3^) were cut to obtain an average of 20 grids, each one including at least 5 cells, which were analyzed along non-serial sections. Motor neurons were selected based on classic morphological features (multipolar cells with dispersed nuclear chromatin and prominent nucleoli). In order to improve the selection of motor neurons, we also applied a size exclusion criterion which is validated by several previous studies adjusted to various mouse strains (Morrison et al. 1998; Martin et al. 2007; Fornai et al. 2008a, b; Ferrucci et al. 2010; Fulceri et al. 2012; Fornai et al. 2014; Natale et al. 2015). This consists of excluding those lamina IX neurons measuring less than 30 μm of maximum diameter, which limits the analysis to phasic alpha-motor neurons (α-MNs). Despite ruling out gamma motoneurons (γ-MNs) and most tonic α-MNs, this allows to rule out type I Golgi projecting neurons.

As far as it concerns muscle samples, we cut little blocks (each measuring a volume of 5 mm^3^) in the central part of the belly at the level of the wider muscle size in order to achieve a homogeneous analysis of the same muscle area in each mouse. Since variations in muscle fibers orientation may lead to different structural perspectives and different measurements, each block was cut following the same longitudinal orientation. This procedure allows keeping constant the reference points while following the course of peripheral nerve fibers within muscle length, thus reducing experimental bias as much as possible. Analysis at TEM was oriented by a previous light microscopy observation of 1–2 μm-thick serial semi-thin sections, which were cut using an ultramicrotome (Porter Blum MT-1 Reichert-Jung). These slices were stained with 1% toluidine blue and 1% methylene blue in 1% sodium tetraborate, and they were analyzed concerning the homogeneity of muscle segments and nerve fiber tracts. Ultrathin sections were stained with uranyl acetate and lead citrate. For TEM analysis, 90 nm-thick sections from both spinal cord and muscle specimens were cut with an ultramicrotome and stained with uranyl acetate and lead citrate. Grids were examined at JEOL JEM-100SX transmission electron microscope (JEOL, Tokyo, Japan) at magnification ranging from 3000× up to 10000×.

### Morphometric analysis of mitochondrial alterations

Mitochondria were defined as altered according to criteria being validated by previous morphological studies (Fornai et al. 2008a, b; Natale et al. 2015) as follows: (1) significantly decreased electron density of the matrix (dilution, vacuolization, cavitation); (2) fragmented and ballooned cristae (intracristal swelling); (3) partial or complete separation of the outer and inner membranes; (4) mitochondrial swelling. Accordingly, the following data were calculated: (1) density of mitochondria in muscle and distal axon of WT and SMA mice; (2) percentage of altered mitochondria in the muscle and distal axon of WT and SMA mice; (3) mitochondrial swelling, assessed by measuring the maximum and minimum mitochondrial diameter in both muscle and distal axons of WT and SMA mice. In order to extend the characterization and the quantification of abnormal mitochondria, we analyzed the occurrence of paracrystalline inclusions (PCIs) within altered mitochondria within the muscle. Mitochondrial PCIs were defined as rigid rectangular crystals which fill most of the mitochondrial volume (Hammersen et al. 1980; Ghadially 1988; Vincent et al. 2016). These electron-dense regular bodies consist of stacked sheets (each one name crystal or filament) with a reciprocal placement which may be either oblique or parallel. The number of mitochondria containing these specific inclusions as ultrastructural disease hallmarks was quantified and expressed as a percentage of total muscle mitochondria of both WT and SMA mice.

### Post-embedding immunoelectron microscopy

Post-embedding immunoelectron microscopy was carried out to test different antibodies in ultrathin sections cut from the same resin-embedded sample block. Ultrathin sections were collected on nickel grids and processed for protein detection after the removal of OsO_4_. As reported in our previous studies (Lenzi et al. 2016; Ferese et al., 2020), this step is recommended for antigen unmasking, while maintaining a good ultrastructural detail. This, in turn, allows a better visualization of immuno-gold particles located within a sharp cell context, which guarantee the count of immuno-gold particles within specific cell compartments. After washing in PBS, the grids were incubated in a blocking solution containing 10% goat serum and 0.2% saponin in PBS for 20 min at 21 °C. Grids were then incubated with the primary antibody solution containing mouse monoclonal anti-SMI-32 antibody (Covance, Emeryville, CA, USA, diluted 1:20) or mouse monoclonal anti-SMN antibody (BD Bioscience, San José, CA, USA, diluted 1:20) with 0.2% saponin and 1% goat serum in PBS in a humidified chamber overnight, at 4 °C. After washing in PBS, grids were incubated with the secondary antibody conjugated with gold particles (20 nm mean diameter, for gold particle anti-mouse, BB International, Treviso, Italy), diluted 1:20 in PBS containing 0.2% saponin and 1% goat serum for 1 h at 21 °C. Control sections were incubated with the secondary antibody only. After rinsing in PBS, grids were incubated with 1% glutaraldehyde for 3 min, they were washed in distilled water to remove traces of salts and prevent precipitation of uranyl-acetate, and they were counterstained with a saturated solution in distilled water of uranyl acetate and lead citrate to be finally observed by using a Jeol JEM SX100 electron-microscope (Jeol, Tokyo, Japan).

### Statistical analysis

For mitochondrial morphometry, values were expressed either using the absolute value or as a percentage of normal numerical distributions. Data are reported as the mean or the mean percentage ± S.E.M. Inferential statistics to compare groups was carried out using Student's *t* test (H_0_ probability was rejected when less than 5%, *P* ≤ 0.05).

## Results

### Muscle fiber architecture and sub-cellular structures are altered in SMA

**Fig. 1:**
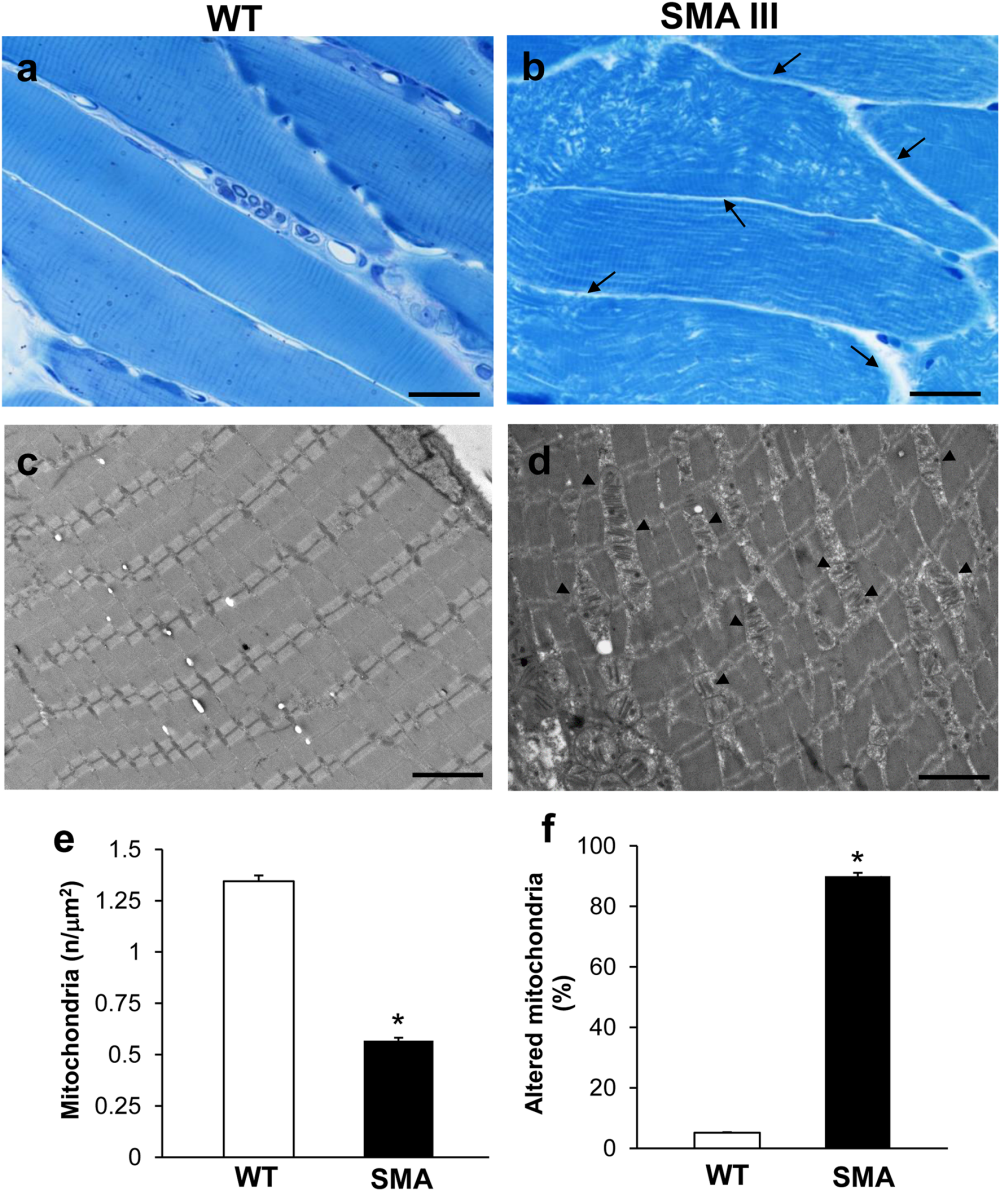
Muscle fiber architecture and sub-cellular structures are altered in SMA compared with WT mice. **a**, **b** Representative pictures at light microscopy of methylene- and toluidine blue-stained semi-thin sections from gastrocnemius muscle in WT and SMA mice. WT mice **a** show a regular, longitudinal, and parallel arrangement of aligned muscle fibers with the typical banding pattern. Nuclei at the periphery of the fibers are well visible. Muscle from SMA mice **b** shows unparalleled and disarranged fibers and myofibrils, with the space among fibers appearing enlarged (arrow). **c**, **d** Representative TEM micrographs of gastrocnemius muscle from WT and SMA mice. WT mice **c** show well-arranged myofibrils with the typical pattern of dark and light bands. No ultrastructural alterations are detected, and a geometrical alignment of sarcomeres is evident. Muscle from SMA mice **d** shows several disarranged areas with a significant increase in intersarcomeric area containing altered, swollen mitochondria (arrowhead). **e**, **f** Graphs report the amount of mitochondria expressed as mitochondrial density (number of mitochondria/μm^2^), and the number of altered mitochondria expressed as a percentage of total mitochondria in SMA and WT mice. Values are the mean number ± S.E.M. from 20 homogenous areas each measuring 6 μm^2^. Scale bar: **a**, **b** = 50 µm; **c**, **d** = 3.5 µm. *P ≤ 0.05 compared with WT.

As shown in representative semi-thin micrographs from the gastrocnemius muscle of WT and SMA mice obtained at light microscopy (Fig. 1a, b), a severe disarrangement of muscle structure in SMA mice occurs compared with the well-aligned (parallel) and normal architecture of muscle fibers in WT mice. In fact, muscle from SMA mice (Fig. 1b) shows unparalleled and disarranged fibers and myofibrils, with an enlarged space among fibers. This contrasts with the longitudinal, regular, and parallel arrangement of aligned muscle fibers showing the typical banding pattern in WT mice, with visible nuclei at the periphery of the fibers (Fig. 1a). In these semi-thin sections, we could dissect the homogeneous segment to be analyzed under TEM (Fig. 1c, d).

When examined at TEM, muscle fibers from SMA mice (Fig. 1c) exhibit a severe loss of the normal sarcomere structure and regular sarcomeres’ alignment, to such an extent that muscle fibers are barely recognizable as composed of sarcomeric units. Instead, the increased distance between vestigial sarcomeres, which can be clearly appreciated by the naked eye in SMA compared with WT mice, is a witness of the severe disarrangement in SMA muscle fibers. This is evident in representative pictures of Fig. 1c, d, respectively. In these same representative pictures, one can also appreciate the occurrence of a few, yet dramatically altered mitochondria within the muscle of SMA compared with WT mice. This was quantified by measuring the density of mitochondria along with the number of altered mitochondria in the muscle. Altered mitochondria were considered those featuring (1) significantly decreased electron density of the matrix (dilution, vacuolization, cavitation); (2) fragmented and ballooned cristae (intracristal swelling); (3) partial or complete separation of the outer and inner membranes; (4) mitochondrial swelling. As shown in the graph of Fig. 1e, mitochondrial density is decreased by roughly twofold in the muscle of SMA compared with WT mice. Remarkably, most of the mitochondria (nearly 90%) detected in the muscle of SMA mice correspond to altered ones, as evident from the graph of Fig. 1f reporting the amount of altered mitochondria in SMA compared with WT mice.

**Fig. 2:**
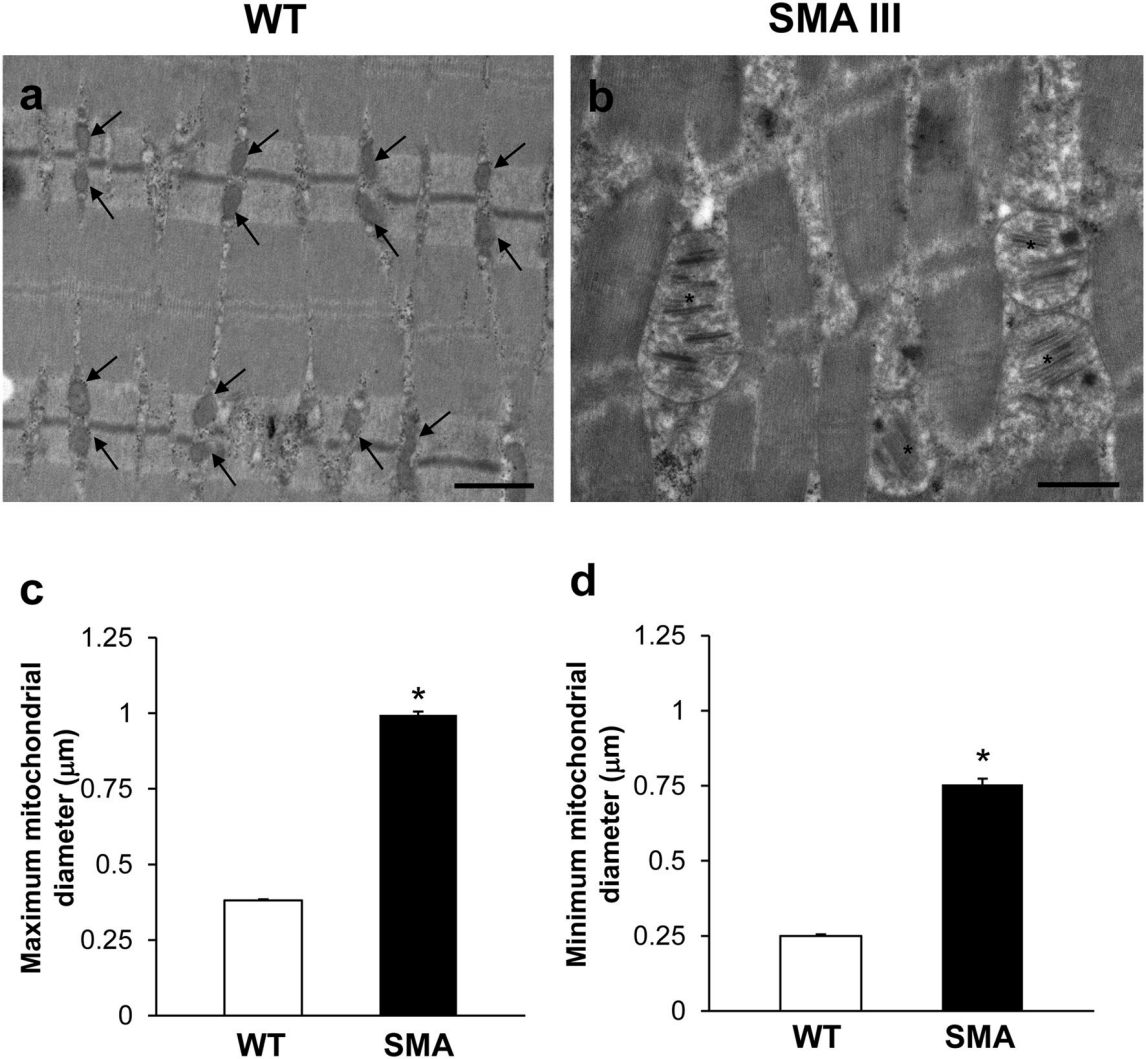
Pathological mitochondrial swelling occurs in the muscle of SMA mice. **a**, **b** Representative TEM micrographs at higher magnification showing mitochondria in gastrocnemius muscle from WT and SMA mice. WT mice **a** show a normal pattern of mitochondria (arrows). SMA mice **b** show altered, swollen mitochondria with enlarged cristae (asterisk). **c**, **d** Graphs report the maximum (**c**) and minimum (**d**) mitochondrial diameter measured within the muscle of SMA compared with WT mice. Values are the mean number ± S.E.M. of 50 mitochondria per mouse (500 mitochondria per group). Scale bar: **a**, **b** = 1 µm. *P ≤ 0.05 compared with WT

In representative pictures obtained at higher magnification (Fig. 2a, b), one can clearly appreciate the dramatic mitochondrial and sarcoplasmic reticulum alterations occurring in the muscle of SMA mice compared with WT mice. In fact, contrarily to the muscle fibers of WT mice (Fig. 2a) containing, well-shaped, healthy mitochondria and sarcoplasmic reticula (concerning both arrangement of the cristae/cisternae, and matrix electron density), muscle fibers of SMA mice (Fig. 2b) are impressively filled with aberrant, extremely swollen mitochondria with enlarged cristae, and sarcoplasmic reticulum with swollen cisternae. In order to quantify pathological mitochondrial swelling, which appears as an increase in mitochondrial size due to a swelling of the mitochondrial structure, ultrastructural morphometry concerning the maximum and minimum mitochondrial diameter was applied. As shown in the graphs of Fig. 2c, d, a dramatic increase in both maximum and minimum mitochondrial diameter was measured in the muscle of SMA compared with WT mice, which is a witness of pathological mitochondrial swelling characterizing SMA muscle.

**Fig. 3:**
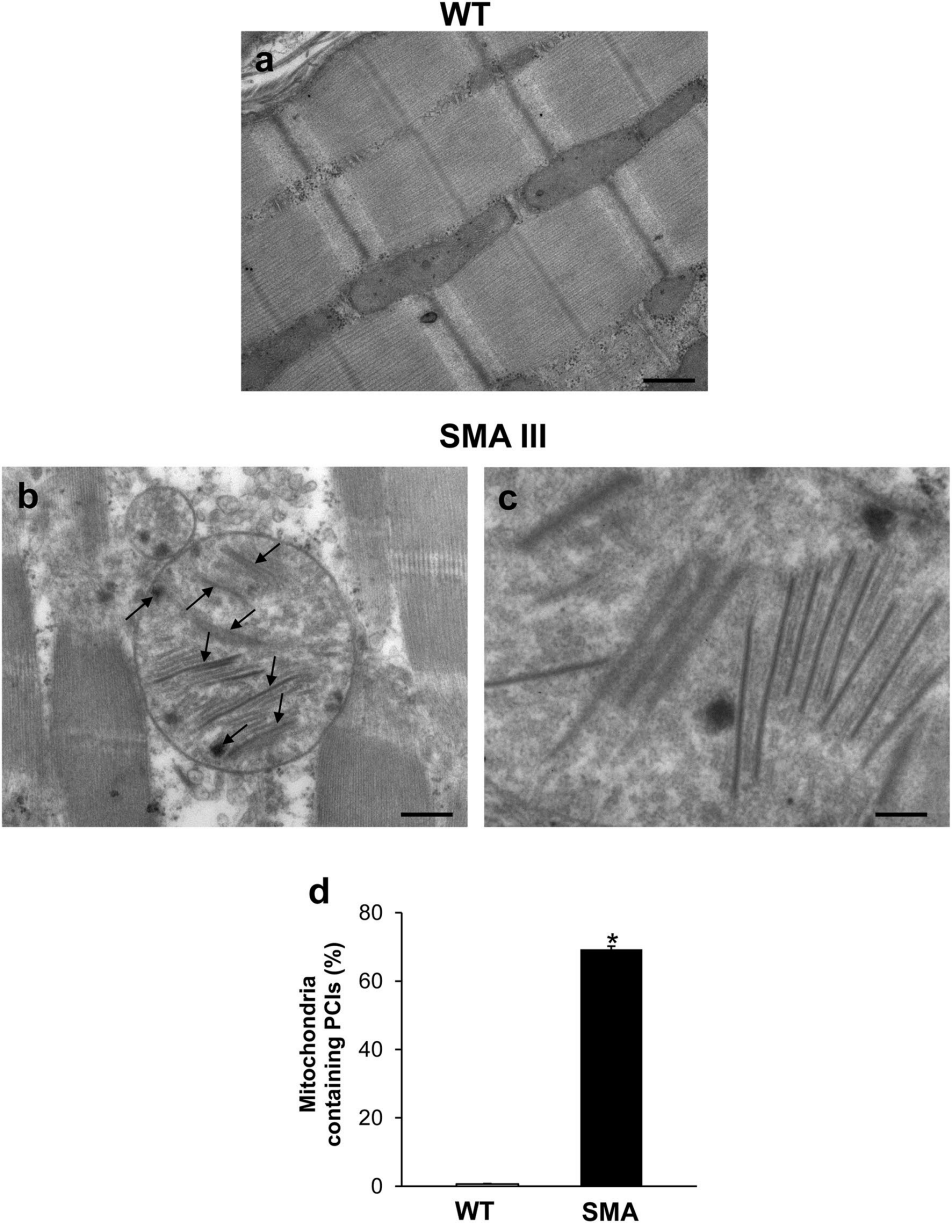
Representative TEM micrographs of mitochondrial paracrystalline inclusions (PCIs) occurring in the muscle of SMA mice. **a**, **b** Representative TEM micrographs showing mitochondrial paracrystalline inclusions (PCIs) in gastrocnemius muscle from WT and SMA mice. While PCIs are totally absent in the WT mouse (**a**), the mitochondria of the muscle from SMA mouse (**b**) were all filled with these rigid, rectangular, and electron-dense crystals (**c**). The graph **d** reports the percentage of mitochondria containing PCIs measured within the muscle of SMA compared with WT mice. Values are the mean percentage ± S.E.M. from 20 homogenous areas each measuring 6 μm^2^. Scale bar: **a**,** b** = 1 µm; **c** = 0.14 µm. *P ≤ 0.05 compared with WT

In order to extend the characterization and the quantification of altered mitochondria within the muscle of SMA compared with WT mice, we analyzed the occurrence of paracrystalline inclusions (PCIs). As reported in representative TEM micrographs (Fig. 3a–c), these mitochondrial inclusions are clearly detectable in the muscle of SMA, while they are totally absent in the WT mice. In fact, as reported in the graph (Fig. 3d), contrarily to the muscle fibers of WT mice, where PCIs were never detected, the mitochondria of the muscle of SMA were all filled with mitochondrial PCIs, which appear as rigid rectangular crystals approximately 500 nm long, and 120 nm wide (Fig. 3c).

### Muscle spindle fibers’ architecture is altered in SMA

**Fig. 4:**
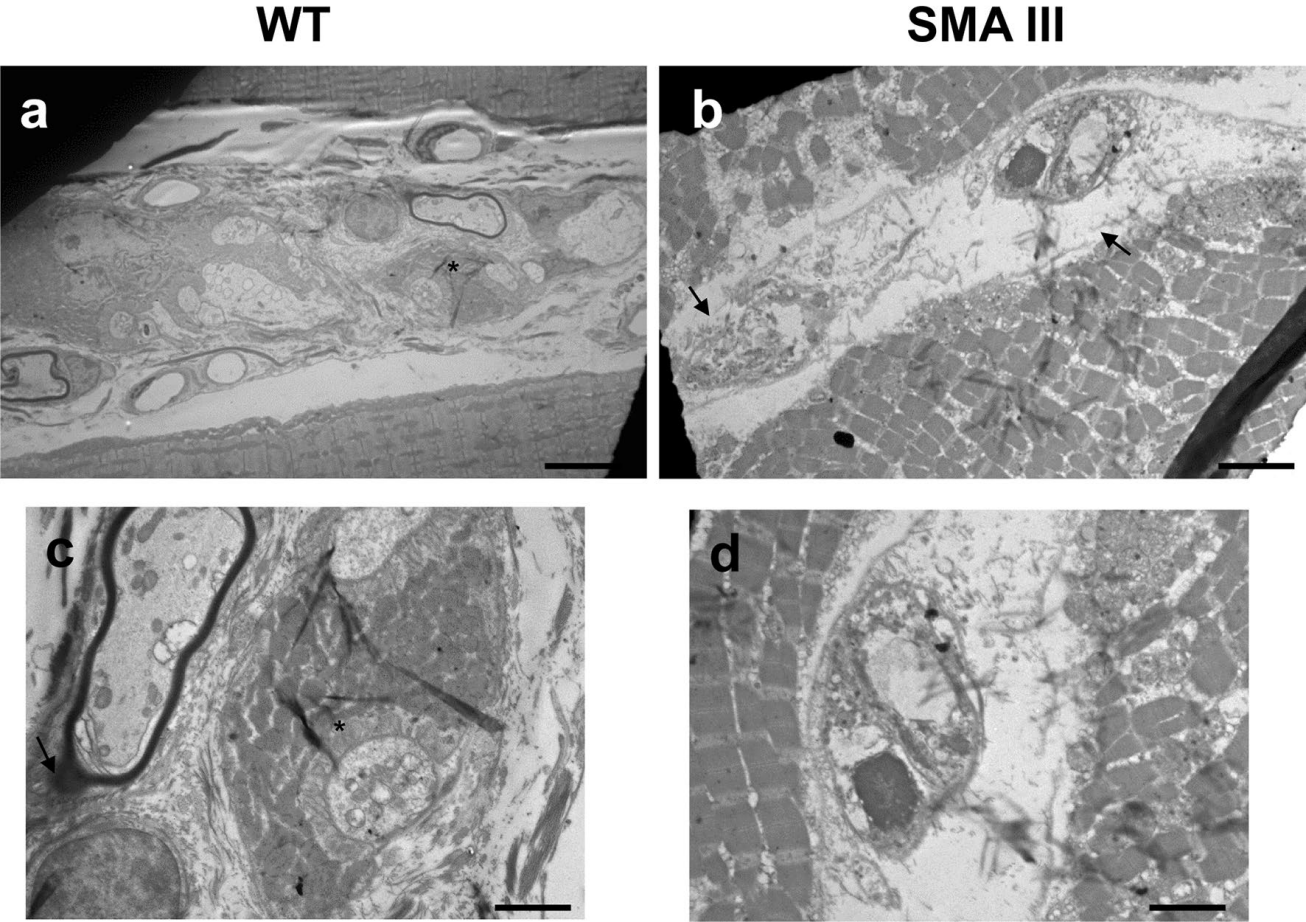
Representative TEM micrographs of muscle spindle from WT and SMA mice. **a**, **c** Representative TEM micrographs from WT mice showing muscle spindles with normal ultrastructure, concerning both intrafusal fibers with well-organized myofilaments, and regular capsule. At higher-magnification micrographs, one can appreciate well-conformed fibers receiving axon terminals (asterisk), which are surrounded by a well-organized layer of sarcoplasm. **b**, **d** Representative TEM micrographs from SMA mice showing dramatically altered muscle spindle architecture. Myofilaments are not recognizable, the capsule is thickened or disintegrated (arrow), and anomalous fibers with completely disarranged surrounding sarcoplasm appear. Scale bar: **a**, **b** = 18 µm; **c**, **d** = 3.5 µm

Compared with WT mice (Fig. 4a, c) the intrafusal muscle fibers from SMA mice (Fig. 4b, d) are dramatically altered to the condition in which myofilaments are not recognizable, and the capsule appears thickened with evident areas where disintegration has begun. At higher-magnification micrographs from WT mice (Fig. 4c), one can appreciate well-conformed fibers receiving axon terminals which are surrounded by a well-organized layer of sarcoplasm. This organization is completely lost in SMA mice, where anomalous fibers with completely disarranged surrounding sarcoplasm appear (Fig. 4d).

### Distal axons feature loss of myelin sheath and clogging in SMA

**Fig. 5:**
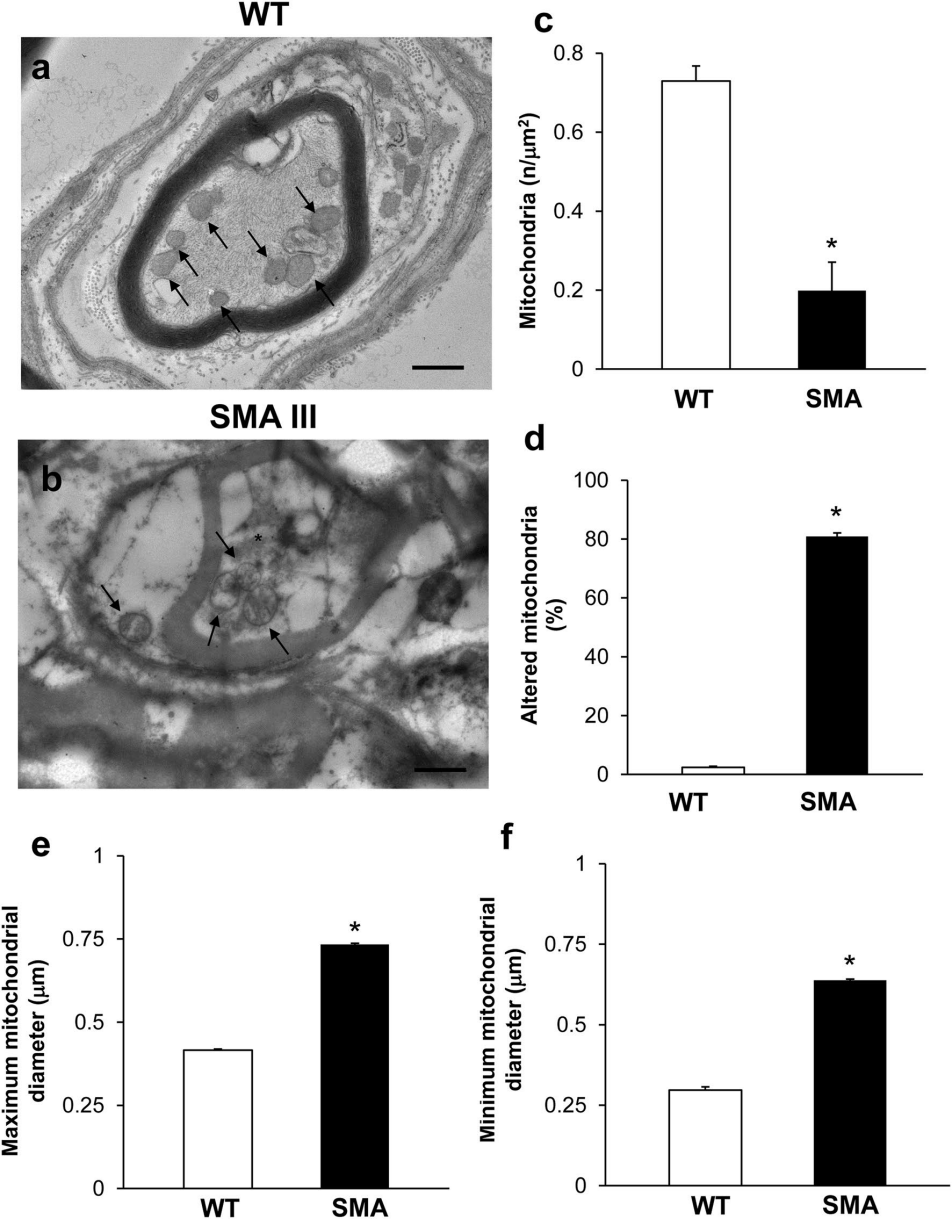
Representative electron micrographs of distal axons in WT and SMA mice. **a** Electron micrograph of cross-sections of myelinated axons from WT mice showing well-confirmed and organized myelinated sheath with axons lacking any obstructive materials within the axoplasm. A well-organized cytoplasm and healthy mitochondria are distinguishable in the axoplasm (arrows). **b** Electron micrograph of cross-sections of myelinated axons from SMA mice. In SMA mice, an abnormal, disrupted myelin sheath occurs, which appears to intrude the axoplasm. Electron-dense, heterogeneous structures, clogging the axonal lumen are present within the axoplasm of distal axons (asterisk), including altered mitochondria (arrow). **c**–**f** Graphs report the number of mitochondria in the distal axons, the percentage of altered mitochondria, and the maximum and minimum mitochondria diameter, respectively. Values are the mean number ± S.E.M. from 20 distal axons per mouse (200 axons per group, **c**, **d**); Values are the mean number ± S.E.M. of 50 mitochondria per mouse (500 mitochondria *per* group, **e**, **f**). Scale bar: **a**, **b** = 1 µm. *P ≤ 0.05 compared with WT

The ultrastructural morphometry of distal axons reveals remarkable alterations consisting of myelin sheath disruption and axon clogging in SMA compared with WT mice. Representative TEM micrographs at high magnification provide a clear perspective of both myelin sheath disruption axonal clogging (Figs. 5a, b). The normal axonal constituents observed in WT mice, including neurofilaments and healthy mitochondria (Fig. 5a), are no longer distinguishable within SMA mice axons (Fig. 5b). The myelin sheath also seems to intrude the axoplasm, aside from it being markedly altered. The axoplasm of distal axons from SMA mice is entirely jammed by abnormal, amorphous structures with enhanced electron-density, including neurofilaments and altered mitochondria. This was confirmed by mitochondrial morphometry indicating a dramatic reduction in the number of mitochondria in SMA mice, most of which correspond to altered mitochondria (graphs of Fig. 5c, d). This was confirmed when assessing mitochondrial swelling by measuring the maximum and minimum mitochondrial diameter, which is dramatically increased in SMA compared with WT mice (graphs of Fig. 5e, f). This witnesses for pathological mitochondrial alterations, which characterize both the muscle and distal axons of SMA mice.

The axonal inclusions observed here, while being largely reminiscent of what we previously described in the G93A mouse model of ALS, are likely to produce deleterious effects concerning the physiology of axonal transport (Magrané et al. 2012, 2014; Natale et al. 2015). Decreased mitochondrial transport associated with cytoskeletal changes and/or impaired removal of altered mitochondria (mitophagy) and biogenesis of novel ones (mitobiogenesis) may similarly contribute to early pathological changes in axons and muscles of ALS and SMA (Wen et al. 2010; Natale et al. 2015; Ripolone et al. 2015; Xu et al. 2016). This may be also due to indirect effects of SMN on mitochondrial function, possibly by affecting the splicing, translation, or mRNA transport of genes required for mitochondrial homeostasis and transport (Acsadi et al. 2009; Natale et al. 2015; Xu et al. 2016).

### Neuromuscular junction (NMJ) architecture is altered in SMA

**Fig. 6:**
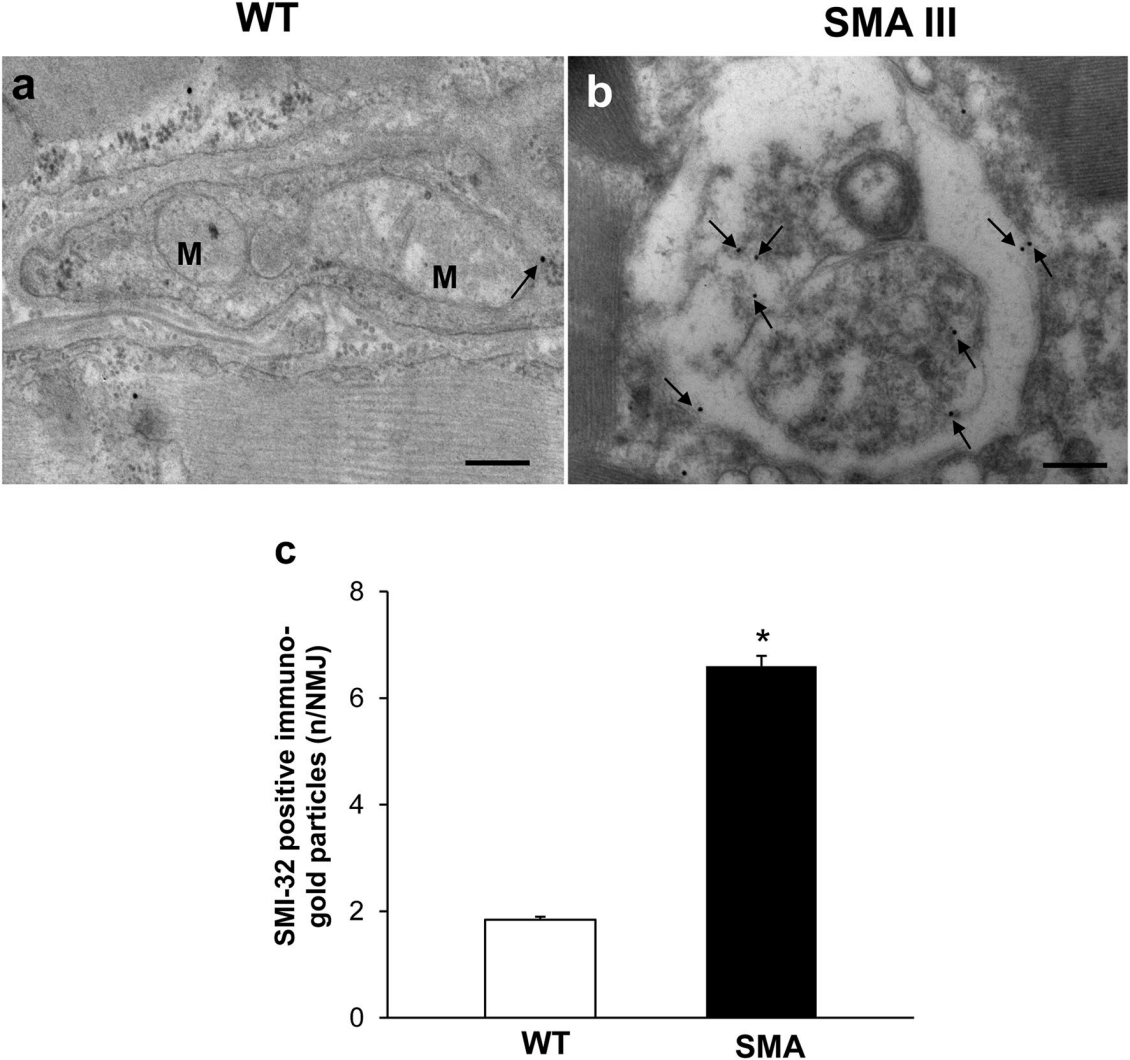
Representative localization of SMI-32 in neuromuscular junction (NMJ) in WT and SMA mice. Electron micrograph from WT mouse **a** showing a normal, well conformed NMJ. SMI-32 immuno-gold particles (arrows) are barely detectable within the a well-organized cytosol. **b** In SMA mouse, the normal NJM architecture is completely lost and sub-cellular compartments are no longer distinguishable, while a strong SMI-32 immuno-gold staining is evident (arrows). The graph **c** reports the number of SMI-32 immuno-gold particles in SMA and WT mice. Values are the mean number ± S.E.M. of 10 NMJ per mouse (100 NMJ per group). Scale bar: **a**, **b** = 0.3 µm. *P ≤ 0.05 compared with WT. *M* mitochondria

**Fig. 7:**
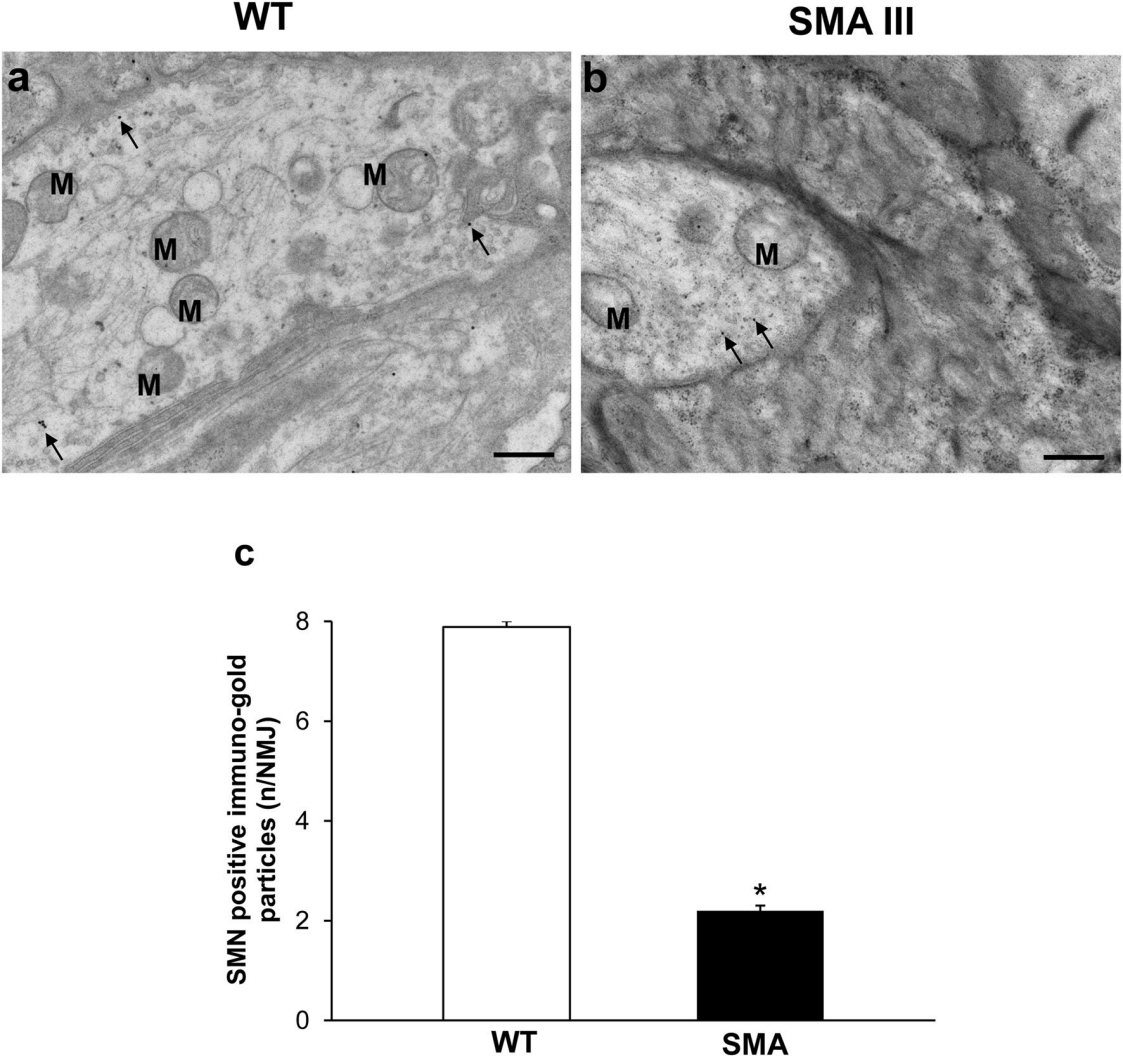
Representative localization of SMN in neuromuscular junction (NMJ) in WT and SMA mice. Electron micrograph of NMJ from WT mouse **a** showing numerous SMN immuno-gold particles (arrows). **b** Only few SMN immuno-gold particles (arrows) are detected in NMJ from SMA mouse. **c** The graph reports the number of SMN immuno-gold particles in SMA and WT mice. Values are the mean number ± S.E.M. of 10 NMJ per mouse (100 NMJ per group). Scale bar: **a**, **b** = 0.6 µm. *P ≤ 0.05 compared with WT. *M* mitochondria

**Fig. 8:**
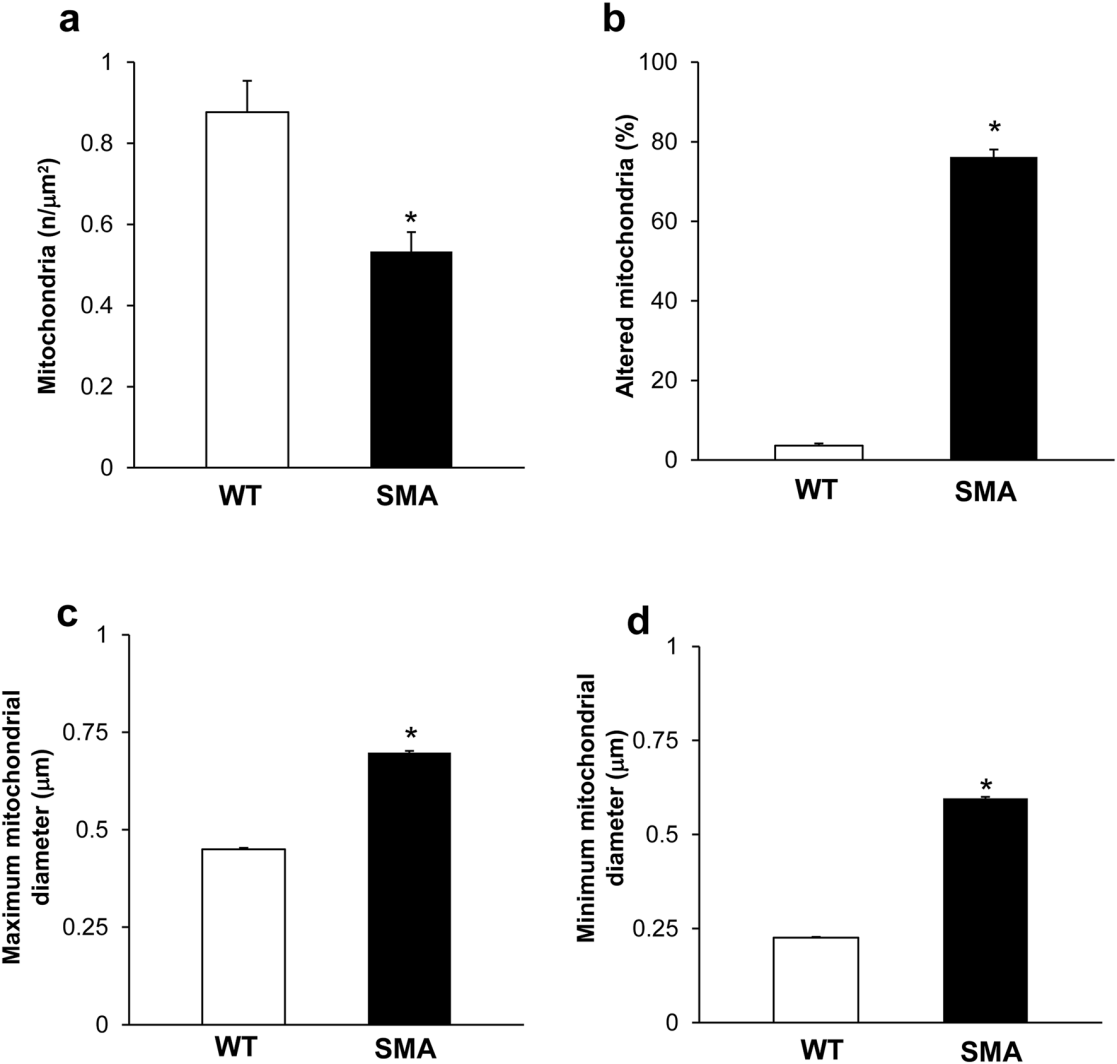
Ultrastructural morphometry of mitochondria in neuromuscular junction (NMJ) in WT and SMA mice. Graphs report the number of mitochondria (**a**), the percentage of altered mitochondria (**b**), and the maximum and minimum mitochondria diameter (**c**, **d**, respectively) in NMJ from WT and SMA mice. Values are the mean number ± S.E.M. from 10 NMJ per mouse (100 NMJ per group, **a**, **c**, **d**); values are the mean percentage ± S.E.M. of 10 NMJ per mouse (100 NMJ per group, **b**). *P ≤ 0.05 compared with WT

The fine structure of NMJ from WT mice appeared well conformed and mitochondria and cytoplasmic structures were distinguishable (Figs. 6a, 7a). In contrast, NMJ from SMA mice featured ultrastructural alterations, which may vary from a slight derangement of cytoplasmic structures up to a completely loss of architecture (Figs. 6b, 7b). This, in turn, further substantiates the remarkable and multiple morphological alterations occurring in SMA III compared with WT. In particular, swollen NMJ showed amorphous material containing vestigial mitochondria and massive neurofilament accumulation, as evidenced by the intense SMI-32 staining (Fig. 6b, c). Conversely, SMN immuno-gold particles were scarcely and randomly placed within NMJ from SMA mice compared with WT (Fig. 7), as expected by this double transgenic mouse model of SMA III, in which SMN protein levels are barely detectable compared with the homozygous strain. When detailing the ultrastructural alterations occurring in NMJ of SMA mice, morphological mitochondrial defects were also evident. In detail, mitochondrial morphometry demonstrated a severe reduction in the number of mitochondria in SMA mice, along with increase in the percentage of altered mitochondria (Fig. 8a, b), as demonstrated by the significant increase both in maximum and minimum diameter (Fig. 8c, d) compared with WT mice.

### The ultrastructure of motor neurons and proximal axons is well- preserved despite SMI-32 accumulation in SMA

**Fig. 9:**
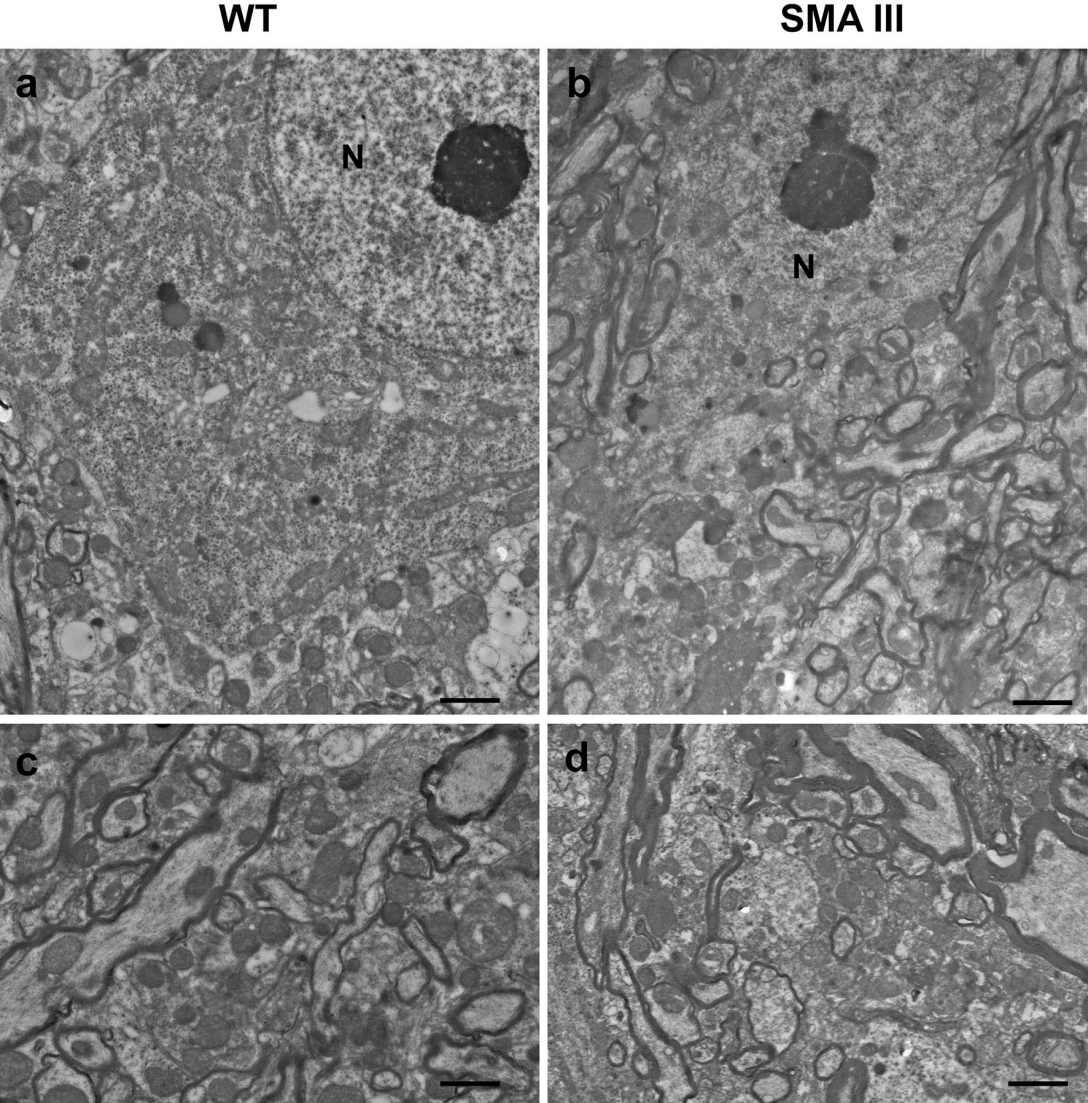
Representative TEM micrographs of the lumbar spinal cord, lamina IX, from WT and SMA mice. **a**, **c** Regular ultrastructure of motor neurons and proximal axons in WT mice, featuring well-evident nucleolus, well-organized myelin sheath, as well as regularly sized and well-shaped mitochondria within the cytoplasm and axoplasm. **b**, **d** Preserved ultrastructure of motor neurons and proximal axons in SMA mice overlapping with that of WT mice, concerning well-organized myelin sheath, and healthy mitochondria within the cytoplasm and axoplasm. Scale bar: **a**, **b** = 5 µm; **c**, **d** = 0.4 µm. *N* nucleus

Surprisingly, the ultrastructure of both motor neurons in the anterior horn and proximal axons from SMA mice (Fig. 9b, d) appears largely preserved, resembling that of WT mice (Fig. 9a, c). Similar to WT mice, the nucleus in the perikarya of SMA mice is not condensed and the nucleolus is well evident (Fig. 9a, b). At higher magnification, one can appreciate how the myelin sheath is well-organized and both perikaryon and axoplasm possess well-shaped and regularly sized mitochondria in both WT and SMA mice (Fig. 9c, d). This contrasts dramatically with the impressive loss of muscle structure and distal axon architectural disruption, including mitochondrial damage, which was observed in these SMA mice. These findings were quite unexpected since the gross morphology of MNs in these same mice, as assessed in our previous studies, appeared largely heterotypic, often enlarged and hyperchromatic, with intensely basophilic cytoplasm and nucleus (Fulceri et al. 2012; Biagioni et al. 2017). Instead, the well-preserved ultrastructure observed here seems to rule out overt pathological changes in spared MNs and proximal axons, contrasting markedly with the dramatic ultrastructural alterations which characterize the muscle, muscle spindle, and distal axons.

**Fig. 10:**
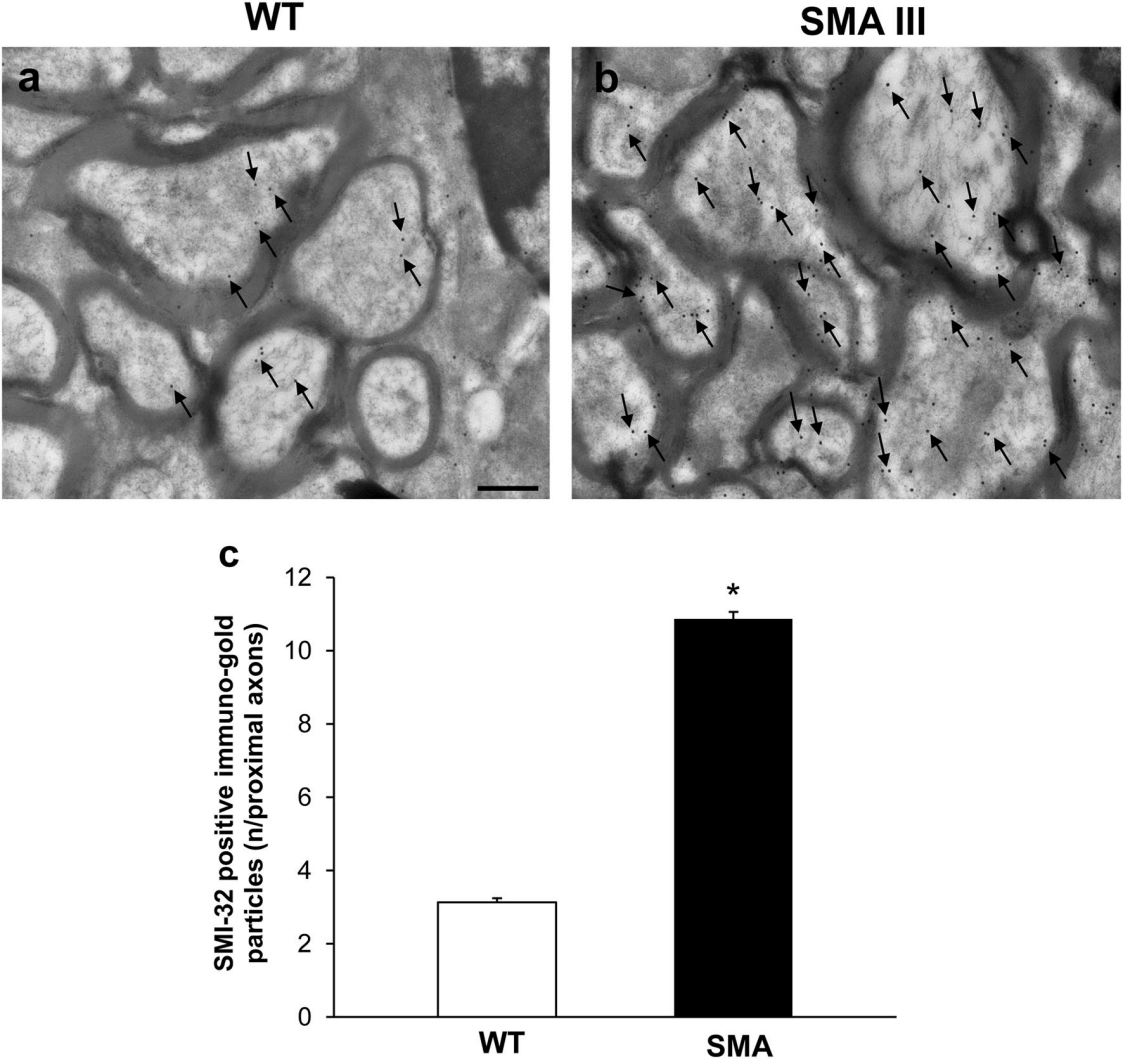
Representative localization of SMI-32 in the ventral root of the lumbar spinal cord from WT and SMA mice. Electron micrograph of cross-section of the ventral root of the lumbar spinal cord from WT mouse **a** showing few SMI-32 immuno-gold particles (arrows). **b** A large amount of SMI-32 immuno-gold particles (arrows) are detected in the ventral root of the lumbar spinal cord from SMA mouse. **c** The graph reports the number of SMI-32 immuno-gold particles in SMA and WT mice. Values are the mean number ± S.E.M. of 200 axons of the ventral root per group. Scale bar: **a**, **b** = 0.2 µm. *P ≤ 0.05 compared with WT

**Fig. 11:**
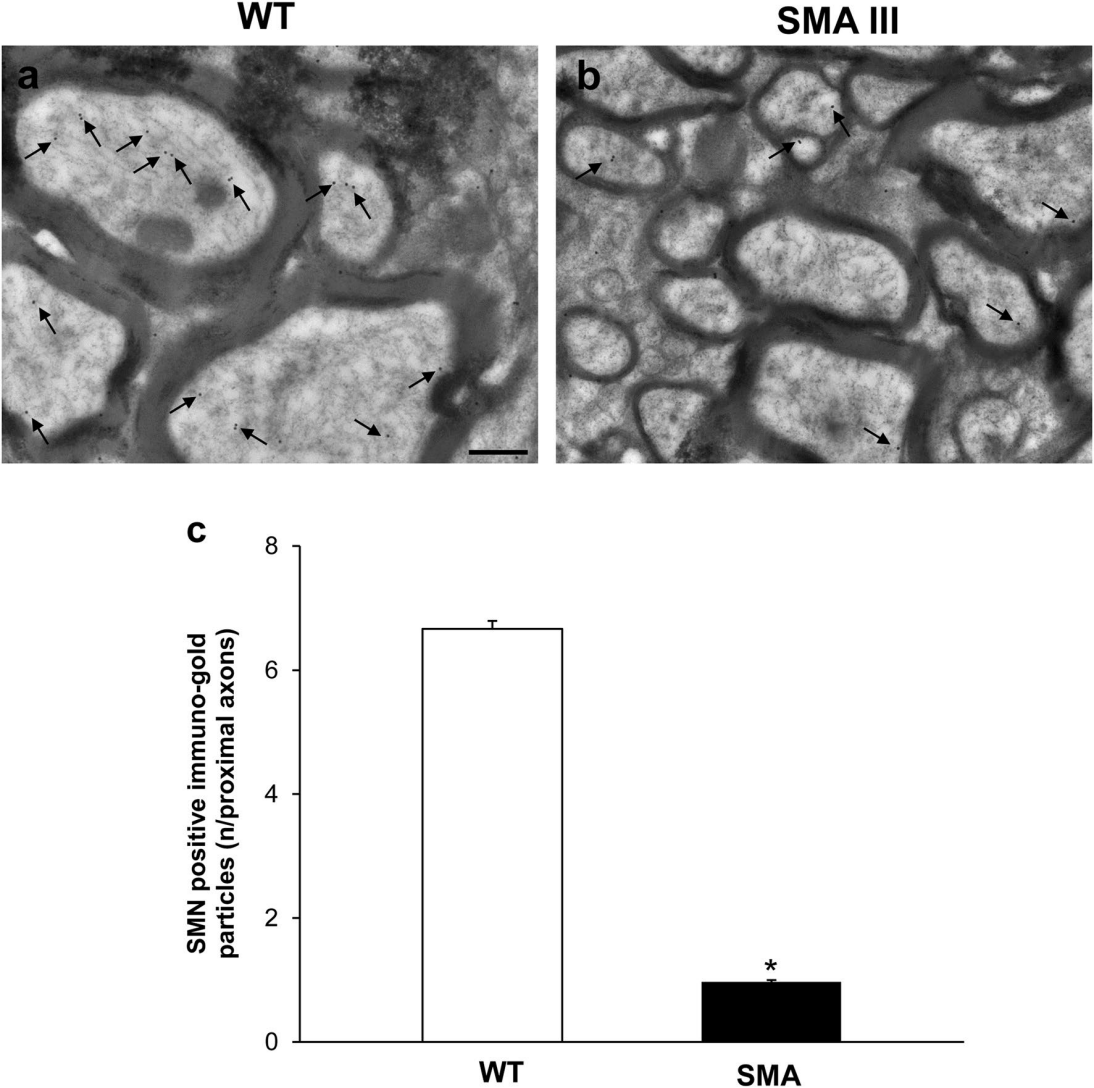
Representative localization of SMN in the ventral root of the lumbar spinal cord from WT and SMA mice. Electron micrograph of cross-section of the ventral root of the lumbar spinal cord from WT mouse **a** showing numerous SMN immuno-gold particles (arrows). **b** Only few SMN immuno-gold particles (arrows) are detected in the ventral root of the lumbar spinal cord from SMA mouse. **c** The graph reports the number of SMN immuno-gold particles in SMA and WT mice. Values are the mean number ± S.E.M. of 200 axons of the ventral root per group. Scale bar: **a**, **b** = 0.2 µm. *P ≤ 0.05 compared with WT

In order to check whether a peripheral clogging may generate an upstream accumulation of neurofilament proteins even in the absence of substantial morphological damage, the ultrastructure of the ventral root was analyzed. In fact, if a peripheral clogging exists this may block the axonal flow. In turn, this is expected to be slowed down even upstream, up to the ventral root. If this is the case, one should expect an accumulation of the SMI-32 protein in SMA III mice, which did not occur in WT. Consistently, we demonstrated that in SMA III mice the amount of SMI-32 protein, even upstream at the level of the ventral root, was higher compared with WT mice (Fig. 10). Of course a reduction in SMN was confirmed even at this level, according to the marked deficiency which is produced in the disease in knockout double transgenic SMA III mouse model (Fig. 11).

## Discussion

In the present study, we provided ultrastructural evidence that is consistent with peripheral denervation in slow progressive SMA. This is confirmed by a disruption of muscle fiber architecture and distal axonal clogging, which occurs in the absence of overt alterations in the ultrastructure of proximal axons and motor neurons in the SMA III mouse model. Again, distal axons feature remarkable alterations in myelin sheath and clogging of axoplasm by abnormal, amorphous structures, including intrusions of the myelin sheath itself, electron-dense material as well as abnormal mitochondria. Based on these observation, one should expect an alteration of nerve conduction velocity (NCV). However, previous electrophysiological studies, both in mouse models and humans, indicate that alteration of NCV is not a feature of type III SMA (Ryniewicz 1977; Schwartz and Moosa 1977; Monani et al. 2003). In fact, as reported by Monani et al. (2003), there is no significant difference between type III SMA mice and age-matched controls when analyzing motor NCVs of the tibial nerve. This may be due to the very distal site (placed within the muscle), which are altered in the nerve. In fact, albeit the conduction velocity was not altered the amplitude of the electric potential at the level of the muscle was significantly decreased. This seems to be confirmed in the present study by a slight morphological alteration in the proximal nerve fibers compared with the extensively altered axoplasm within muscle nerve terminals. Remarkable ultrastructural alterations also occur in the muscle spindles of these mice, especially concerning intrafusal fibers and nerve terminals, whereby the normal architectural organization is completely disrupted compared with WT mice. Our data are in line with evidence indicating that neuromuscular disorders feature an early, peripheral axonopathy involving sensory and/or motor fibers, whereby the communication between the muscle and central nervous system is no longer effective. The dramatic clogging of distal axons by aberrant mitochondria and amorphous structures documented here along with the remarkable mitochondrial alterations quantified within the muscle of SMA mice are a witness of potential defects in transport mechanisms, which are known to occur in both ALS and SMA (Wen et al. 2010; Natale et al. 2015; Xu et al. 2016; Limanaqi et al. 2017).

In line with our observations, a general consensus is now emerging that NMJ breakdown is an early event in SMA pathogenesis, which may contribute to muscle denervation, progressive MN loss, and motor symptom onset. This is bound to (1) the lack of SMN protein, which besides MNs, is critical for the homeostasis of NMJ synapses, sensory and motor axons, and muscles, and/or, (2) impairment of retrograde signals or transport mechanisms from NMJs (Rajendra et al. 2007; Bowerman et al. 2012; Bottai and Adami 2013; Boido and Vercelli 2016). Consistent with our morphological evidence on muscle and peripheral axonal changes, several alterations have been described at the peripheral level in both SMA models and patients, which can be crucial players of MNs alterations. In SMA animal models, earliest detectable pathological changes are observed at the NMJs and muscle, which are followed, only at later time points, by motor neuronal loss (Mutsaers et al. 2011; Sleigh et al. 2011; Ling et al. 2012; Fayzullina and Martin 2014). The mechanisms through which NMJ defects may contribute to MNs loss and motor symptoms onset include a variety of phenomena. These consist of impaired release of neurotrophic factors, neurofilament buildup, and poor axonal sprouting, reduced terminal arborization, disruption in synaptic vesicle release, aberrant expression of synaptic proteins, delayed post-synaptic maturation, muscle denervation, defects in motor neuron excitability due to altered Ca^2+^ homeostasis, and loss of Schwann cells leading to defects in endplate remodeling and nerve-directed maturation of acetylcholine receptor (AChR) clustering (Cifuentes-Diaz et al. 2002; Jablonka et al. 2006, 2007; Rajendra et al. 2007; Kariya et al. 2008; Kong et al. 2009; Ling et al. 2010, 2012; Ruiz et al. 2010; Torres-Benito et al. 2011; Murray et al. 2012; Shababi et al. 2014).

Aberrant ultrastructure of NMJs and delayed maturation of myotubes have also been reported in human prenatal specimens from SMA fetuses (Martinez-Hernandez et al. 2009, 2013). Features of neurogenic atrophy are also evident from skeletal muscle biopsy in SMA I patient (Thirunavukkarasu et al. 2020). Remarkably, within muscles from SMA I-III patients, mitochondrial damage and impaired mitochondrial biogenesis have been documented (Ripolone et al. 2015). This is evident by the occurrence of a few, altered mitochondria reminiscent of our present observations. Again, SMA patient-derived muscle cells and MNs from induced pluripotent stem cells (iPSCs) are impaired to form NMJs due to AChR clustering defects (Arnold et al. 2004; Yoshida et al. 2015). Thus, formation and maintenance of NMJs and muscle may precede the occurrence of MN death in SMA, which suggests that the vulnerability of MNs is due to both autonomous cell susceptibility to various stresses, and even early peripheral defects contributing first to muscle denervation and then, a loss of MNs (Fidziańska and Rafalowska 2002; Fischer et al. 2004; Wadman et al. 2012).

Similar to what was reported for ALS models, early abnormalities in the sensory, mostly proprioceptive circuit, play a key role in determining altered MN excitability, up to MN loss, and motor system defects in SMA models (Jablonka et al. 2006; Ling et al. 2010; Mentis et al. 2011; Imlach et al. 2012; Lalancette-Hebert et al. 2016; Fletcher et al. 2017; Limanaqi et al. 2017). In detail, MN loss in SMA mice follows afferent synapse loss with a precise temporal and topographical pattern (Mentis et al. 2011). At early disease stages, SMA motor neurons show reduced proprioceptive reflexes that correlate with decreased number and function of synapses on motor neuron innervating proximal hindlimb muscles and medial motor neurons innervating axial muscles. At later time points, this extends to motor neurons innervating distal hindlimb muscles. This may be partly related to SMN deficiency in proprioceptive synapses, though the precise molecular mechanisms remain to be elucidated (Fletcher et al. 2017).

The neuromuscular reflex arc, or gamma loop, which integrates the proprioceptive information for muscle length and activity to modify motor neuron output and muscle contraction, is emerging as a key to understanding neuromuscular deficits in diseases such as ALS and SMA (Limanaqi et al. 2017; Vukojicic et al. 2019). The sensory portion of the arc is composed of proprioceptive neurons and fibers, which convey information from the equatorial region of intrafusal fibers of the muscle spindle towards α-MNs within the ventral horn of the spinal cord. The polar regions of intrafusal fibers are instead innervated by γ-MNs that regulate intrafusal fiber stretch so that they retain proper tension and sensitivity during muscle contraction or relaxation. This is seminal to maintain the sensitivity of proprioception during dynamic muscle activity and to prevent muscular damage. It is remarkable that, in contrast to γ-MNs being largely spared in murine models of both ALS and SMA, α-MNs and proprioceptive fibers seem to be mostly vulnerable (Lalancette-Hebert et al. 2016; Powis and Gillingwater 2016; Falgairolle and O’Donovan 2020). This is not surprising if one considers that contrarily to γ-MNs, α-MNs receive direct, monosynaptic input from proprioceptive fibers, which may be early affected in neuromuscular disease. Thus, impaired proprioception is expected to reduce α-MN firing ability, with a compensatory increase of γ-MN firing, which may, in turn, contribute to deficits in muscle contraction and limb movement (Fletcher et al. 2017; Lalancette-Hebert et al. 2016; Limanaqi et al. 2017). At present, it cannot be ruled out that the marked ultrastructural alterations that we observe at the distal axonal level in the SMA III mouse, may apply to sensory, besides motor fibers, which would require dedicated studies to be confirmed. However, this may be the case if one considers that impaired proprioception is also associated with an altered muscle spindle morphology, which is indeed documented here for the first time in SMA III, confirming what previously described in both ALS and SMA I (Kararizou et al. 2006; Limanaqi et al. 2017; Kröger and Watkins 2021). Morphological and morphometric evidence from SMA I patients’ biopsies indicates that the muscle spindle may be a critical player in the pathophysiology of SMA (Kararizou et al. 2006). In detail, the overall ultrastructure and the area of the muscle spindle, along with the diameter of the intrafusal fibers, and the mean area of nuclei of the intrafusal fibers are reduced, while the thickness of the capsule is increased in SMA patients compared with controls (Kararizou et al. 2006). This overlaps with what we observed here in the SMA III mice, whereby intrafusal fibers are altered to the condition in which myofilaments are not recognizable, and the capsule appears thickened with evident areas of disintegration.

In conclusion, our observations confirm a seminal role for peripheral alterations in SMA pathogenesis, shifting the ultimate anatomical target in type III SMA from the spinal cord to the muscular endplate. Obviously, our morphological data call for further molecular studies which are expected to confirm potential triggers in SMA pathogenesis. As a future perspective, immunocytochemical studies investigating SMN occurrence in peripheral compartments besides MNs may be key to unravel any potential correlations between SMN levels and disease progression. This may apply beyond SMA since SMN protein levels are critical in other neuromuscular disorders including ALS (Veldink et al. 2005). In this frame, it would be interesting to investigate the disease-modifying effects of compounds, which are known to increase SMN levels while preventing both motor neuron loss, and motor impairment associated with mitochondriopathy and axon clogging in ALS and SMA models (Feng et al. 2008; Fornai et al. 2008a, b; Harahap et al. 2012; Natale et al. 2015; Biagioni et al. 2017).

## Declarations

### Data availability statement

Data supporting reported results can be found in an on-line repos-itory which can be provided upon request to the corresponding author.
